# Worldwide Innovative Network (WIN) Consortium in Personalized Cancer Medicine: Bringing next-generation precision oncology to patients

**DOI:** 10.18632/oncotarget.28703

**Published:** 2025-03-12

**Authors:** Wafik S. El-Deiry, Catherine Bresson, Fanny Wunder, Benedito A. Carneiro, Don S. Dizon, Jeremy L. Warner, Stephanie L. Graff, Christopher G. Azzoli, Eric T. Wong, Liang Cheng, Sendurai A. Mani, Howard P. Safran, Casey Williams, Tobias Meissner, Benjamin Solomon, Eitan Rubin, Angel Porgador, Guy Berchem, Pierre Saintigny, Amir Onn, Jair Bar, Raanan Berger, Manon Gantenbein, Zhen Chen, Cristiano de Pádua Souza, Rui Manuel Vieira Reis, Marina Sekacheva, Andrés Cervantes, William L. Dahut, Christina M. Annunziata, Kerri Gober, Khaled M. Musallam, Humaid O. Al-Shamsi, Ibrahim Abu-Gheida, Ramon Salazar, Sewanti Limaye, Adel T. Aref, Roger R. Reddel, Mohammed Ussama Al Homsi, Abdul Rouf, Said Dermime, Jassim Al Suwaidi, Catalin Vlad, Rares Buiga, Amal Al Omari, Hikmat Abdel-Razeq, Luis F. Oñate-Ocaña, Finn Cilius Nielsen, Leah Graham, Jens Rueter, Anthony M. Joshua, Eugenia Girda, Steven Libutti, Gregory Riedlinger, Mohammed E. Salem, Carol J. Farhangfar, Ruben A. Mesa, Bishoy M. Faltas, Olivier Elemento, C.S. Pramesh, Manju Sengar, Satoru Aoyama, Sadakatsu Ikeda, Ioana Berindan-Neagoe, Himabindu Gaddipati, Mandar Kulkarni, Elisabeth Auzias, Maria Gerogianni, Nicolas Wolikow, Simon Istolainen, Pessie Schlafrig, Naftali Z. Frankel, Amanda R. Ferraro, Jim Palma, Alejandro Piris Gimenez, Alberto Hernando-Calvo, Enriqueta Felip, Apostolia M. Tsimberidou, Roy S. Herbst, Josep Tabernero, Richard L. Schilsky, Jia Liu, Yves Lussier, Jacques Raynaud, Gerald Batist, Shai Magidi, Razelle Kurzrock

**Affiliations:** ^1^Worldwide Innovative Network (WIN) Association – WIN Consortium, Chevilly-Larue, France; ^2^Legorreta Cancer Center at Brown University, Providence, RI 02912, USA; ^3^Avera Cancer Institute, Sioux Falls, SD 57105, USA; ^4^Ben-Gurion University of the Negev, Be'er Sheva, Israel; ^5^Centre Hospitalier du Luxembourg, Luxembourg; ^6^University of Luxembourg, Esch-sur-Alzette, Luxembourg; ^7^Luxembourg Institute of Health, Luxembourg; ^8^Department of Medical Oncology, Centre Léon Bérard, Lyon, France; ^9^University of Lyon, Claude Bernard Lyon 1 University, INSERM 1052, CNRS 5286, Centre Léon Bérard, Cancer Research Center of Lyon, Lyon, France; ^10^Jusidman Cancer Center, Sheba Medical Center, Ramat Gan, Israel; ^11^Faculty of Medical and Health Sciences, Tel Aviv University, Tel Aviv, Israel; ^12^Fudan University Shanghai Cancer Center, Shanghai, China; ^13^Molecular Oncology Research Center, Barretos Cancer Hospital, Barretos, Brazil; ^14^Life and Health Sciences Research Institute (ICVS), School of Health Sciences, University of Minho, Braga, Portugal; ^15^I.M Sechenov First Medical State University, Moscow, Russian Federation; ^16^INCLIVA Instituto de Investigación Sanitaria, Valencia, Spain; ^17^American Cancer Society, Atlanta, Georgia, MD 21742, USA; ^18^Burjeel Medical City (BMC), Mohamed Bin Zayed City, Abu Dhabi, UAE; ^19^Medical Oncology Deparment. Institut Català d'Oncologia. Oncobell Program (IDIBELL), Universitat de Barcelona (Campus Bellvitge), CIBERONC, Barcelona, Spain; ^20^Sir H.N. Reliance Foundation Hospital and Research Centre, Mumbai, India; ^21^ProCan, Children’s Medical Research Institute, The University of Sydney, Australia; ^22^National Center for Cancer Care and Research Hamad Medical Corporation, Doha, Qatar; ^23^Oncology Institute Ion Chiricuta, Cluj, Romania; ^24^King Hussein Cancer Center, Amman, Jordan; ^25^Instituto Nacional de Cancerología (INCan), Mexico City, Mexico; ^26^Rigshospitalet, Copenhagen, Denmark; ^27^The Jackson Laboratory, The Maine Cancer Genomics Initiative, Bar Harbor, ME 04609, USA; ^28^The Kinghorn Cancer Centre, St Vincent’s Hospital, Darlinghurst, Australia; ^29^School of Clinical Medicine, Faculty of Medicine and Health, University of New South Wales, Sydney, Australia; ^30^Rutgers Cancer Institute, New Brunswick, NJ 08901, USA; ^31^Wake Forest University Health Sciences/Atrium Health (WFUHS), Winston-Salem, NC 27157, USA; ^32^Weill Cornell Medical College, NY 10065, USA; ^33^Tata Memorial Centre, Affiliated to Homi Bhabha National Institute, Mumbai, India; ^34^Institute of Science Tokyo Hospital, Tokyo, Japan; ^35^University of Medicine and Pharmacy Iuliu Hatieganu, Cluj, Romania; ^36^Academy of Medical Sciences, Bucharest, Romania; ^37^Vyas Cancer Research (VCR Park), Maharanipeta, Visakhpatnam, Andhra Pradesh, India; ^38^Cure51, Paris, France; ^39^CHAIM Medical Resource Organization, NY 10950, USA; ^40^Cancer is an A*, LLC, Manalapan Township, NJ 07726, USA; ^41^TargetCancer Foundation, Cambridge, MA 02139, USA; ^42^Vall d’Hebron Hospital Campus and Institute of Oncology (VHIO), Barcelona, Spain; ^43^The University of Texas, M.D. Anderson Cancer Center, Houston, TX 77030, USA; ^44^Yale School of Medicine, New Haven, CT 06510, USA; ^45^The University of Chicago, Chicago, IL 60637, USA; ^46^The University of Utah, Salt Lake City, UT 84112, USA; ^47^Segal Cancer Centre, Jewish Hospital, McGill University, Montreal, Quebec, Canada; ^48^Medical College of Wisconsin, Milwaukee, WI 53226, USA

**Keywords:** precision oncology, N-of-1 basket trials, AI algorithms, digital pathology, drug access

## Abstract

The human genome project ushered in a genomic medicine era that was largely unimaginable three decades ago. Discoveries of druggable cancer drivers enabled biomarker-driven gene- and immune-targeted therapy and transformed cancer treatment. Minimizing treatment not expected to benefit, and toxicity—including financial and time—are important goals of modern oncology. The Worldwide Innovative Network (WIN) Consortium in Personalized Cancer Medicine founded by Drs. John Mendelsohn and Thomas Tursz provided a vision for innovation, collaboration and global impact in precision oncology. Through pursuit of transcriptomic signatures, artificial intelligence (AI) algorithms, global precision cancer medicine clinical trials and input from an international Molecular Tumor Board (MTB), WIN has led the way in demonstrating patient benefit from precision-therapeutics through N-of-1 molecularly-driven studies. WIN Next-Generation Precision Oncology (WINGPO) trials are being developed in the neoadjuvant, adjuvant or metastatic settings, incorporate real-world data, digital pathology, and advanced algorithms to guide MTB prioritization of therapy combinations for a diverse global population. WIN has pursued combinations that target multiple drivers/hallmarks of cancer in individual patients. WIN continues to be impactful through collaboration with industry, government, sponsors, funders, academic and community centers, patient advocates, and other stakeholders to tackle challenges including drug access, costs, regulatory barriers, and patient support. WIN’s collaborative next generation of precision oncology trials will guide treatment selection for patients with advanced cancers through MTB and AI algorithms based on serial liquid and tissue biopsies and exploratory omics including transcriptomics, proteomics, metabolomics and functional precision medicine. Our vision is to accelerate the future of precision oncology care.

## INTRODUCTION

Cancer is a leading cause of morbidity and mortality worldwide, with rising incidence including among younger individuals [1]. In 2022, nearly 22 million new cases of cancer were reported, along with 9.7 million cancer-related deaths [2]. Despite significant advances in cancer diagnosis and treatment, the disease remains a major public health challenge around the world. One of the key factors contributing to the complexity of cancer is its diverse molecular landscape, which can vary not only between different types of cancer but also within cancer types and even within individual tumors [3]. As a result, personalized precision approaches to cancer diagnosis and treatment have become increasingly important in recent years [4–10]. While significant strides have been made in understanding cancer biology and developing treatments, there remains a substantial need for further advances in personalized and precise approaches to cancer diagnosis and treatment [11].

Traditional cancer treatment strategies often rely on histopathological assessments and clinical parameters to guide diagnosis and therapy selection. However, the heterogeneity within and between tumors, especially those in the advanced/metastatic state, necessitates a more nuanced understanding of each patient’s unique molecular profile to optimize treatment outcomes. Recent advances in molecular biology and high-throughput technologies have enabled the generation of large-scale genomic, transcriptomic, proteomic, immunomic, and epigenomic data from cancer patients. These molecular profiling techniques have provided valuable insights into the underlying molecular mechanisms driving cancer development and progression, as well as potential therapeutic targets [12–16]. Despite the wealth of data generated by these techniques, translating this information into clinically actionable insights remains a significant challenge. This is due in part to the complexity and heterogeneity of cancer [17], as well as the limitations of existing methods for analyzing and interpreting large-scale molecular data which is usually available on a limited number of patients. There is a need for advanced computational tools and algorithms capable of integrating data from multiple sources, including omics and clinical data, as well as functional assays and response to treatment. Effectively incorporating these data sources into clinical decision-making requires sophisticated data processing approaches [18]. Leveraging artificial intelligence (AI) algorithms to integrate and analyze these diverse datasets holds immense promise for advancing personalized cancer diagnosis and treatment [19]. Importantly, operationalizing a new generation of trials, with designs that are necessarily novel to address the wealth of new data revealed by advanced molecular technologies, is also critically important. The WIN global consortium is ready to take up the challenge by bringing the best possible Precision Oncology trial to patients.

### About WIN

**Figure 1:**
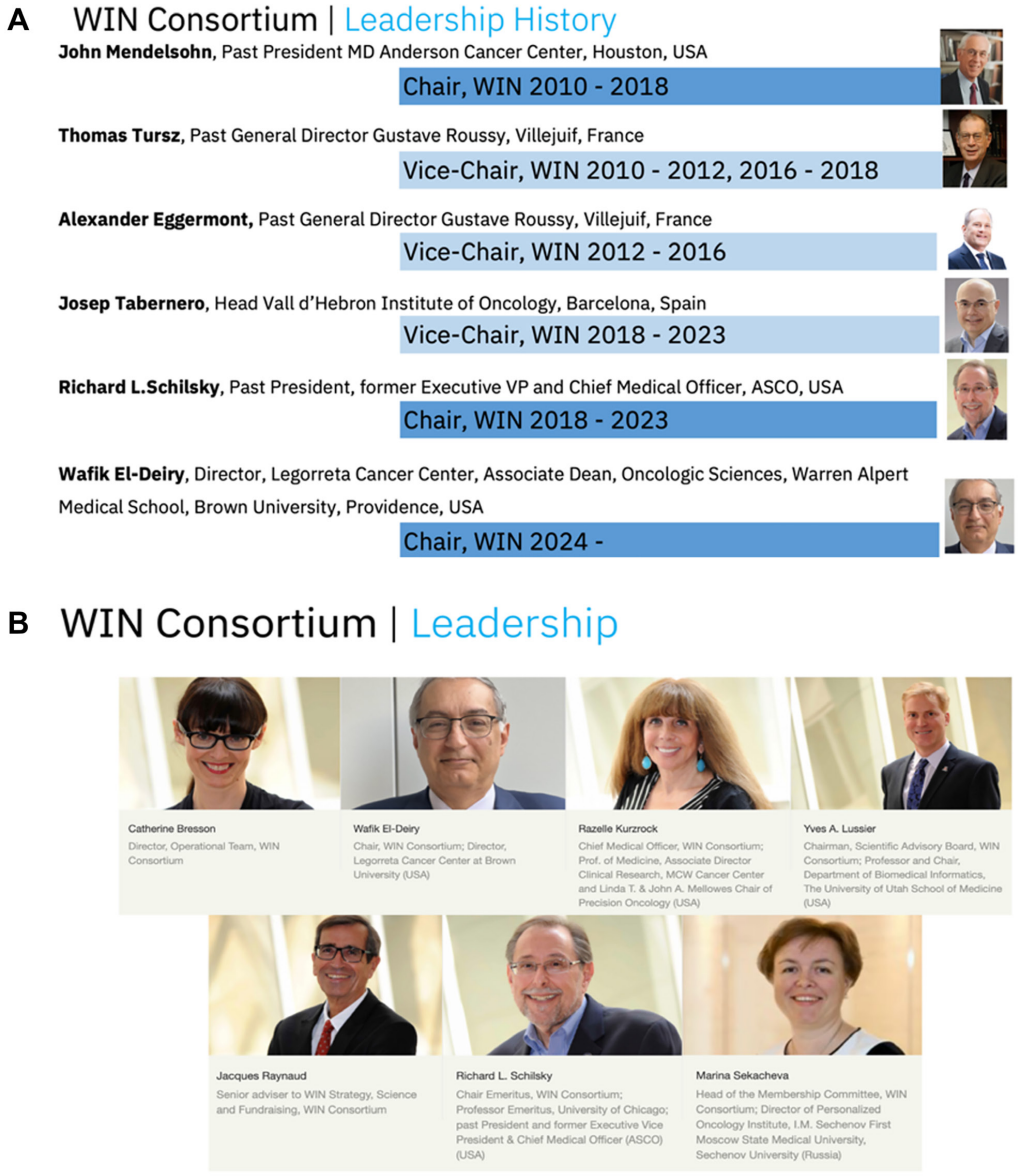
History and Leadership of WIN Consortium in 2024. (**A**) Leadership History of WIN Consortium, (**B**) Current WIN Consortium leadership.

The WIN consortium comprises nearly forty (and growing) academic, industry, research institutions and patient advocates across 18 countries and five continents. It was founded in 2010 in France as a unique non-profit non-governmental organization by the late Dr. John Mendelsohn (Past-President, MD Anderson Cancer Center) and Dr. Thomas Tursz (Past-Director, Gustave Roussy) with subsequent leadership by Dr. Richard L. Schilsky (Past-President and former Chief Medical Officer, ASCO) and Dr. Josep Tabernero (Director of Oncology, Vall D’Hebrón Institute of Oncology) (Figure 1).

WIN was formed on the premise that we can accomplish more together than each organization can achieve working alone and has therefore brought together stakeholders and key opinion leaders in novel precision-based approaches to improve cancer treatment on a global scale.

The WIN consortium is a nimble collaborative global non-profit organization performing complex clinical trials at multiple sites across the world that address a diversity of populations [20]. WIN’s strategy has been to perform proof-of-concept cancer trials designed to explore novel strategies to improve oncology therapeutics and early diagnosis. It has to date completed several novel investigator-led precision oncology studies (including *Investigational Device Exemption (IDE)* [8] and Investigational New Drug (IND) [21] studies) and shared progress over 15 years at convened international precision oncology symposia and high-impact publications [8, 21–27]. WIN is guided by an expert Scientific Advisory Board (SAB) of key opinion leaders in precision cancer medicine from WIN member institutions and non-members: Prof. Yves Lussier, Chair of the SAB, University of Utah School of Medicine, USA; Dr. Stanley Hamilton, Vice-Chair of the SAB, City of Hope Medical Center, California, USA; Dr. Roy S. Herbst, Yale School of Medicine, USA; Prof. Paul Hofman, Nice Sophie Antipolis University, France; Dr. John Quackenbush, Harvard T.H Chan School of Public Health, USA; Prof. Eytan Ruppin, National Institute of Health (NIH) Bethesda, USA; Dr. Lillian Siu, Princess Margaret Hospital, Canada; Prof. Ioana Berindan-Neagoe, University of Medicine and Pharmacy Iuliu Hatieganu, Cluj, Romania; Dr. Anton Buzdin, I.M Sechenov First Medical State University, Russian Federation; Dr. Benedito A. Carneiro, Legorreta Cancer at Brown University, USA; Prof. Gideon Rechavi, Sheba Medical Center, Israel; Dr. Gerald Batist, Segal Cancer Centre, Jewish Hospital, McGill University, Canada.

### WIN symposia

**Figure 2:**
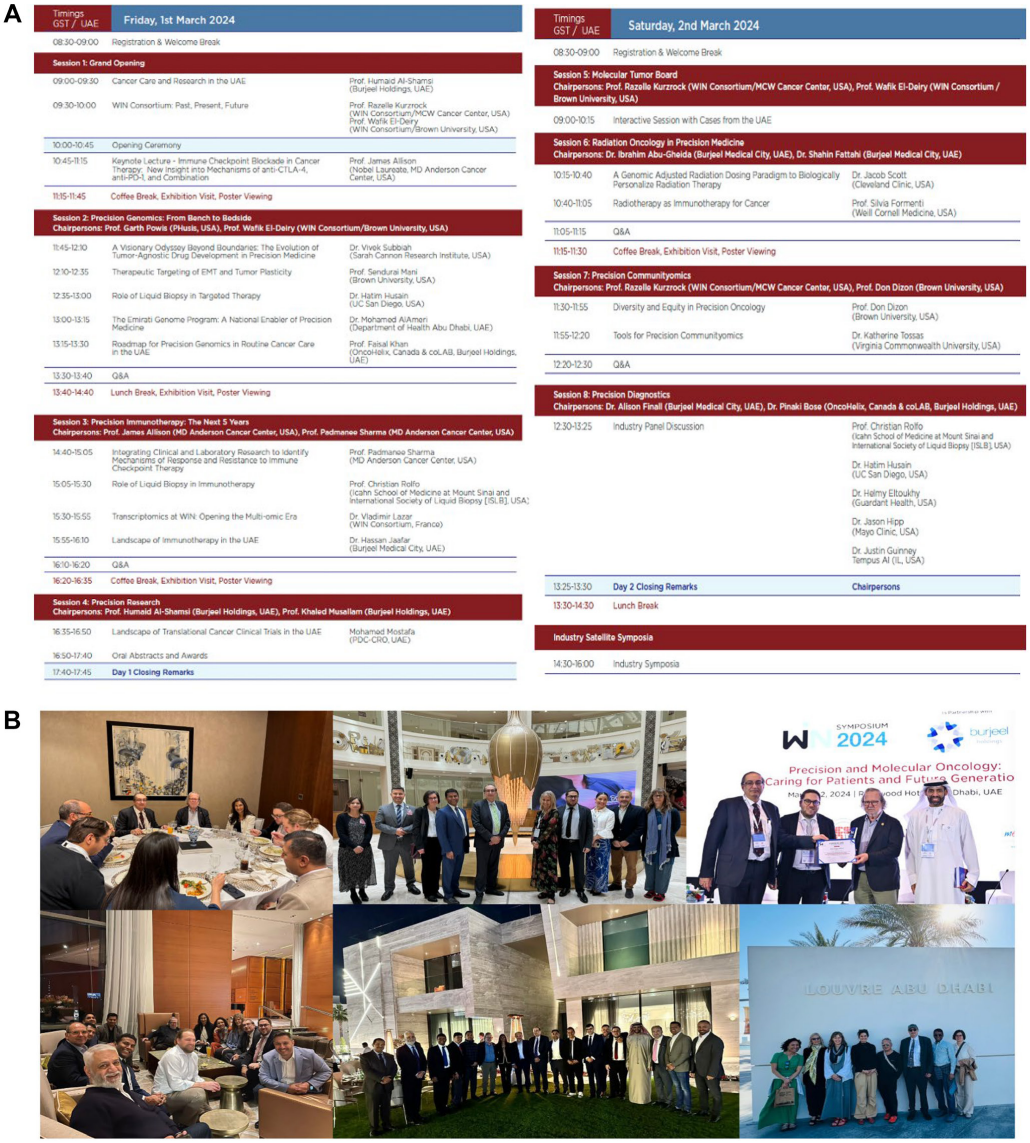
March 2024 WIN symposium held in Abu Dhabi, UAE. (**A**) Scientific program, (**B**) selected photos of the March 2024 WIN Symposium held in Abu Dhabi, UAE. The WIN Symposia bring together an international faculty focused on precision oncology. In addition to the official Symposium events, several social and cultural experiences were enjoyed by participants. The interactions among leaders in the field foster new collaborations around the world.

Regular high-quality educational WIN Symposia have been organized with world leaders in cancer research and precision oncology including Nobel laureates Prof. James P. Allison (2015 and 2024) and Prof. Jennifer Doudna (2019). WIN Symposia have enabled candid discussion across a spectrum of cancer care stakeholders and continuing collaboration with partnering institutions (Figure 2). The most recent WIN Symposium Program from March 2024 (https://winconsortium.org/files/WIN_SYMPOSIUM_2024_PROGRAM_BOOK.pdf) was held in Abu Dhabi, UAE and additional information with abstracts is available in a publication [28].

### WIN Molecular Tumor Board (MTB)

**Figure 3:**
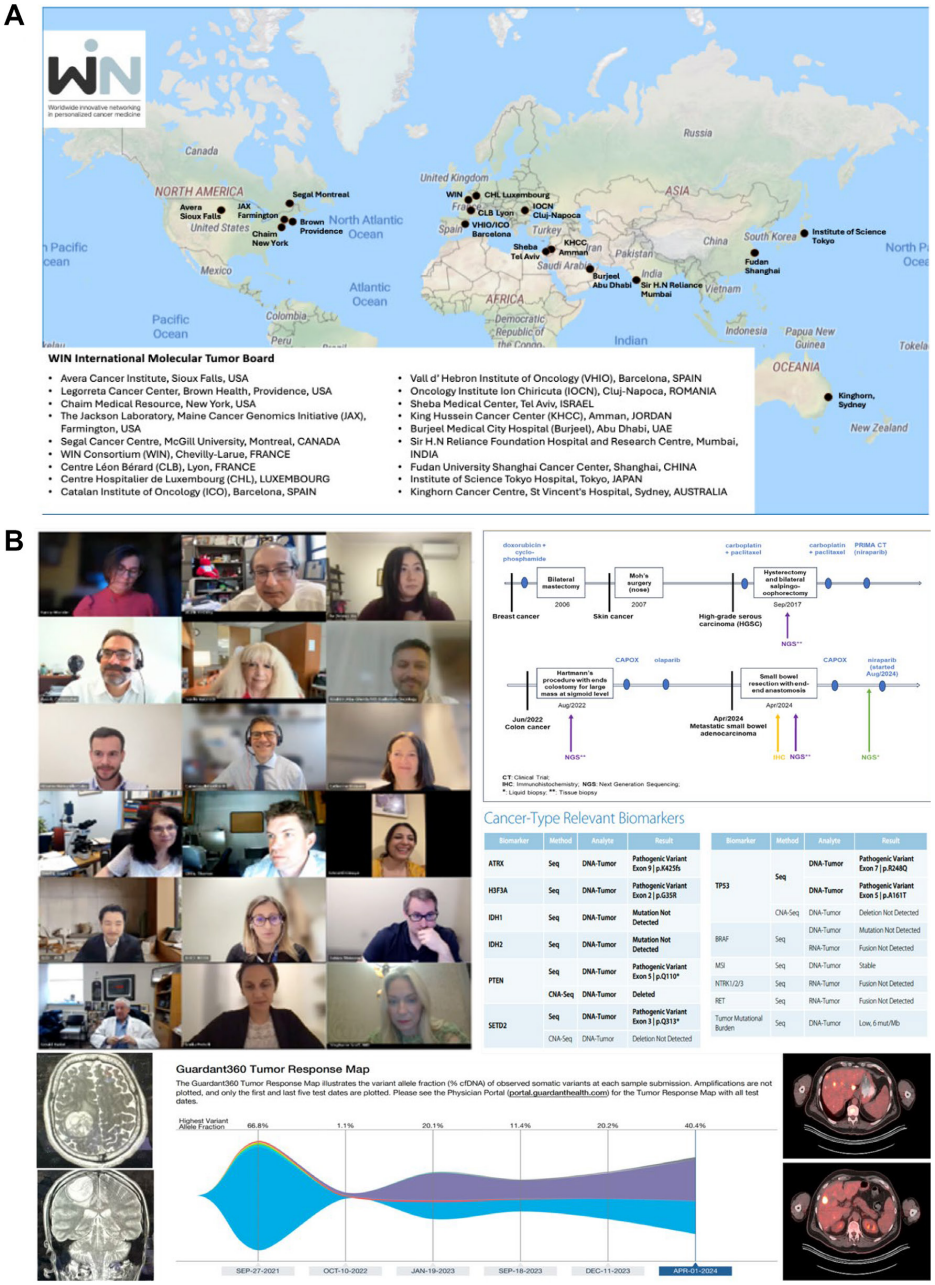
The WIN Consortium has an active international molecular tumor board (MTB). (**A**) with input from experts from all over the world, making this an international effort. (**B**) WIN’s MTB convenes virtually as well as during WIN Symposia (including in Barcelona, 2022 and Abu Dhabi, 2024) and reviews complex cases with molecular profiles to discuss the best personalized therapy for each patient. All cases are discussed after patients provide written informed consent that includes the sharing of deidentified data among WIN members and that allows publication. In 2024, several patients chose to attend the MTB and heard and/or participated in the discussion. Over the past year, the MTB has held more than 20 discussions, covering cases involving colorectal cancer, glioblastoma, rectal cancer, lung cancer, lymphoma, breast cancer, skin cancer, and CNS.

The WIN consortium currently convenes the only academic international Molecular Tumor Board (MTB) chaired/co-chaired by Drs. Razelle Kurzrock (Chief Medical Officer of WIN) and Wafik S. El-Deiry (Chair of WIN) that provides in-depth multidisciplinary input into complex advanced cancer cases incorporating patient, multi-omics and treatment factors into therapeutic recommendations (Figure 3 and Table 1).

**Table 1:** The WIN Consortium international molecular tumor board (MTB) leadership

**Chair**	Dr. R. Kurzrock	WIN Consortium, France Medical College of Wisconsin, USA
**Co-chair**	Prof. W. S. El-Deiry	WIN Consortium, France Legorreta Cancer Center at Brown University, USA
**Co-chair**	Prof. S. Ikeda	Institute of Science Tokyo Hospital, Japan
**Co-chair**	Prof. R. Berger	Sheba Medical Center, Israel
**Co-Chair Co-Moderator for Experimental Therapeutics**	Dr. S. Limaye	Sir HN Reliance Foundation Hospital and Research Centre, India
**Co-Moderator for Early-Phase Trials**	Dr. B. Carneiro	Legorreta Cancer Center at Brown University, USA
**Co-Moderator for Melanoma**	Dr. M. Hadfield	
**Co-Moderator for Breast Cancer**	Dr. S. L. Graff	
**Co-Moderator for Gynaecology Oncology**	Prof. D.S. Dizon	
**Co-Moderator for Lung Cancer**	Dr. C. G. Azzoli	
**Co-Moderator for Molecular Pathology**	Dr. L. Cheng	
**Co-Moderator for Radiation Oncology**	Dr. I. Abu-Gheida	Burjeel Medical City, UAE
**Community Co-Moderator**	Dr. J. Rueter	The Jackson Laboratory, Maine CGI, USA
**Advisor**	Dr. C. Williams	Avera Cancer Institute, USA
**Advisor**	Prof. P. Saintigny	Centre Léon Bérard, France
**Advisor**	Prof. X. Hu	Fudan University Shanghai Cancer Center, China
**Advisor**	Dr. H. Abdel-Razeq	King Hussein Cancer Center, Jordan
**Advisor**	Dr. J. Liu	Kinghorn Cancer Centre, St Vincent’s Hospital, Australia
**Advisor**	Dr. M. Basik	Segal Cancer Center, McGill University, Canada
**Advisor**	Dr. I. Braña	Vall d’Hebron Institute of Oncology, Spain

WIN’s MTB leadership is constituted by both precision oncology and disease experts to guide the discussion.

### WINTHER N-of-1 trial

WIN’s landmark study is the international N-of-1 WINTHER trial funded under the European Union Seventh Framework Program (FP7/grant agreement n°306125), ARC Foundation for Cancer Research (France) and Pfizer Oncology; results were published in Nature Medicine [8]. The study opened a new era in Precision Oncology by utilizing transcriptomic in addition to genomic data, for the first time in the world, to guide N-of-1 treatment selection. The WINTHER trial also introduced for the first time the investigation of normal tissue of origin of the cancer, to make gene expression data of an individual patient more accurate and reduce noise, and an algorithm ranking drugs by predicted efficacy. While the WINTHER trial did not meet its primary end-point of a 1.5× increase in PFS2/PFS1 in 50% of the patients, the trial demonstrated a 1.3× increase in PFS2/PFS1 in 30% of the patients. The goal was perhaps too ambitious at the time given the limitations in the field including lack of availability of drugs for many drivers of cancer, and it would be expected that this could improve in the future.

Importantly, WIN demonstrated, via the WINTHER trial, the ability to operationalize and conduct an international trial, which involved five countries from North America, Europe and the Middle East, working collaboratively [8, 29]. Unique aspects included that the trial was patient-investigator centered with the ability to get the best drugs in the right combination as determined by the WIN MTB to the patients at the right time, efficiently. Typically, pharmaceutical industry-sponsored trials are much less able to deliver precision therapy to multiple cancer drivers as they are usually working with individual molecules. From a clinical standpoint, the study demonstrated improvements in progression-free survival (PFS) and overall survival (OS) in heavily pre-treated patients who received high matching score therapies, where matching scores were derived from the algorithm for predicting efficacy, as compared to those with lower match scores.

Under the current leadership of its new Chair since election in December of 2023, Prof. Wafik S. El-Deiry, MD, PhD, FACP, WIN is growing globally and engaging with the cancer research and oncology communities in a collaborative manner throughout the world to expand the global footprint of the organization and offer diversity of patients for future clinical trials. Building upon the WINTHER trial [8], deployment of novel algorithms for interpreting genomic and transcriptomic results have been developed by the group [22, 23] and ongoing efforts to establish personalized N-of-1 precision oncology trials with a focus on combinations, enrolling globally amongst the WIN membership are underway. By the end of 2024, the WIN Consortium has added 13 new members over the past year. New members’ applications consisting in a questionnaire are submitted to the approval of the WIN governance bodies. The annual membership fee level varies depending upon the member category (industry, small and medium enterprise, nonprofit organizations or academic institutions) and the size of the member for academic institutions. Members are invited to participate in governance bodies and committees including in leadership positions upon election time (with most mandates for a two-year term). Members are encouraged to submit project proposals or participate in WIN projects but have no obligation to do so.

### WIN committees

To facilitate progress towards its mission, in 2024 WIN began establishing a number of Committees including a Data Science Committee, a Biomarkers Committee, a Regulatory Issues in Precision Oncology Committee, a Novel Agents Committee, a Publications Committee, and several Disease Committees including Thoracic Malignancies, GI Cancer, GU Malignancies, CNS Cancer, Breast Cancer, GYN Cancer, and Rare Cancers. Additional WIN Consortium Committees at the planning stage include Radiation Oncology, Digital Pathology, Artificial Intelligence in Precision Oncology, and Functional Precision Medicine.

The Data Science Committee is discussing data platforms and harmonization, data sharing issues, the role of AI in precision oncology, grant opportunities, and guidance publications. At its meetings on June 14, 2024, and September 30, 2024, chaired by Dr. Gerald Batist at Segal Cancer Centre, the Common Database Committee as it was known at the time developed some important agenda items for future progress.

**Figure 4:**
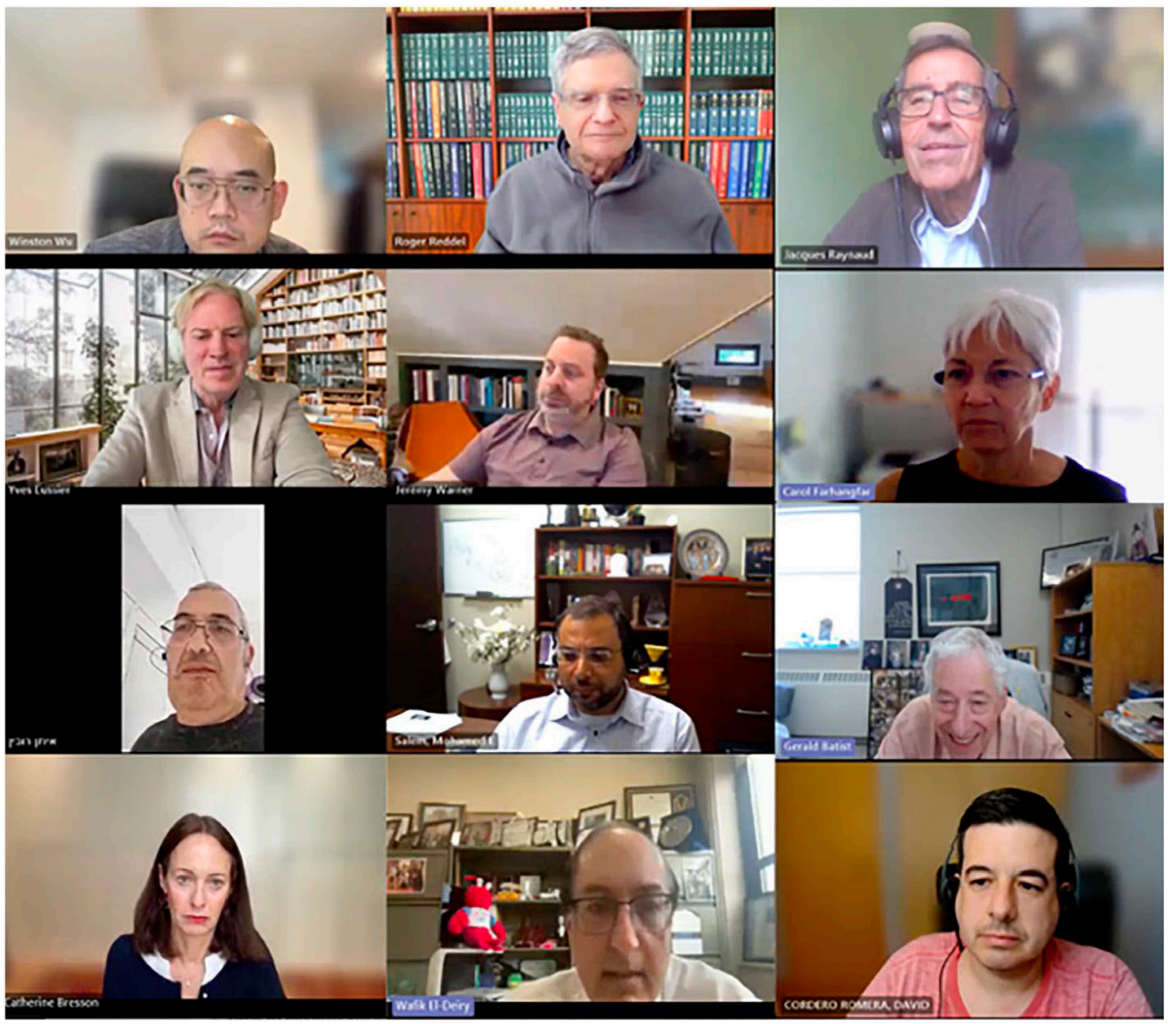
Win consortium common database committee meeting on June 14, 2024.

At the June 14, 2024, WIN Consortium Common Database Committee meeting (Figure 4 and Table 2), it was acknowledged that (1) common representation of the data is important and use of a simple international standard such as OHSDI was recommended. It was also agreed that (2) data protection laws and regulations must be taken into consideration as they are also changing and becoming stricter around the world, (3) specific consent from patient is needed, outlining exactly what would be done with the data (sharing etc.), (4) data of interest includes treatment, outcome, and should be sequential, (5) liquid biopsies should be included, (6) there is need to identify gaps in data that can improve different kinds of AI models, (7) having all the data in one location is preferable in terms of computation with large datasets, (8) partnering in a noncompetitive environment would be ideal. Share how to lift technical barriers, (9) transformation between different data structures/models is very complex, costly and time consuming. However, conversion modalities from different standards do exist (using corporate partners), (10) some of the WIN institutions have already invested resources to do transformation to one type of standard, (11) that WIN would consider innovative technologies which are exploratory and how they will be captured in our database, and (12) there would be further discussions about what platform WIN should work with to integrate datasets.

**Table 2:** WIN consortium common database committee participants

Participants	Institutions
Chair: Dr. Gerald Batist	Segal Cancer Centre, Jewish Hospital, McGill University, Canada
Prof. Yves Lussier	The University of Utah, School of Medicine, USA Chair of WIN Consortium Scientific Advisory Board
Dr. Tobias Meissner	Avera Cancer Institute, USA
Dr. Benjamin Solomon	
Dr. Casey Williams	
Dr. Eitan Rubin	Ben-Gurion University of the Negev, Israel
Prof. Jeremy Warner	Brown University, USA
Dr Brenda Rubenstein	
Dr. Khaled Musallam	Burjeel Medical City, UAE
Dr. Juan Martin-Liberal	Catalan Institute of Oncology (ICO), Spain
Dr Conxi Lázaro	
Dr. David Cordero Romera	
Winston Wu	Exactis, Canada
Prof. Zhen Chen	Fudan University Shanghai Cancer Center, China
Xiaoyi Shu	
Luciane Sussuchi	Fundacao Pio XII – Hospital Cancer de Barretos, Brazil
Dr. Cristiano de Pádua Souza	
Dr. Marina Sekacheva	I.M Sechenov First Medical State University, Russian Federation
Dr. Jia Liu	Kinghorn Cancer Centre, St Vincent’s Hospital, Australia
Dr. Anthony Joshua	
Prof. Roger Reddel	ProCan, Children’s Medical Research Institute, Australia
Dr. Adel Aref	
Dr. Raanan Berger	Sheba Medical Center, Israel
Dr. Sewanti Limaye	Sir H.N. Reliance Foundation Hospital and Research Centre, India
Dr. C.S. Pramesh	Tata Memorial Hospital, India
Prof. Ioana Berindan-Neagoe	University of Medicine and Pharmacy Iuliu Hatieganu, Romania
Dr. Alejandro Piris	Vall d’ Hebron Institute of Oncology, Spain
Dr. Rodrigo Dienstmann	
Dr. Carol Farhangfar	Wake Forest University Health Sciences (WFUHS), USA
Dr. Mohamed Salem	
Prof. Wafik S. El-Deiry	Chair, WIN Consortium, France Legorreta Cancer Center at Brown University, USA
Dr. Razelle Kurzrock	Chief Medical Officer, WIN Consortium, France
Jacques Raynaud	Advisor, WIN Consortium, France
Catherine Bresson	WIN Consortium, France
Fanny Wunder	WIN Consortium, France
Shai Magidi	WIN Consortium, France

Other WIN Consortium Committees were established in 2024. The Biomarkers Committee is concerned with novel tissue agnostic biomarkers, biomarker platforms and technologies, opportunities with industry, and grant opportunities. The Regulatory Issues in Precision Oncology Committee is concerned with FDA guidance on N-of-1 trials, access to off-label approved drugs, reimbursement of drugs used off-label, industry support for N-of-1 trials, and funding for Precision Oncology research. The Novel Agents Committee is focused on novel cancer treatment modalities, novel drug classes, novel cancer therapy agents, novel combinations, support for early-phase Precision Oncology Trials, and grant opportunities. The Publications Committee discusses the status of current publications, plans new publications in precision oncology as well as trials WIN is involved with, and plans for meeting abstracts. A WIN Symposium Organizing Committee works with collaborators and stakeholders in planning WIN’s International Symposia. WIN consortium members based in 18 different countries and 5 continents endeavour to meet during the WIN symposia. However, the regular governance and committee meetings are held under the video conference format (and were held in teleconferences prior to the video conference era). All the meetings are exclusively held in English.

### Rationale for N-of-1

Regarding combination therapies that can be used in precision oncology, it is important to note that the vast majority of advanced/metastatic cancers have multiple co-drivers in a background of tumor and host heterogeneity. With ~300 drugs in oncology, there are ~45,000 two-drug combinations and ~4.5 million three-drug combinations; it will take thousands of years to conduct Phase Ib combination studies across tumor types and sub-types and even longer to perform prospective randomized clinical trials of novel drug combinations. As such, the N-of-1 tumor agnostic (including rare cancers) basket trial precision oncology paradigm that individualizes treatment, including combinations, beyond a main driver, makes sense going into the future. Some compelling data comes from patient advocacy groups that deal with rare cancers in particular, in addition to 15 years of accumulating experience from N-of-1 trials.

It is expected that in the future patients with cancer will be optimally treated according to precision oncology algorithms. The N-of-1 design, particularly when the MTB’s decision takes into consideration an algorithmic matching score, aims to offer a more personalized, precise, and data-driven treatment approach compared to standard-of-care or physician choice alone. By tailoring treatments to individual patient characteristics with input from pharmacogenomics, tumor ‘omics’ with incorporation of real-time feedback including functional organoid and *in vivo* assays, this method has the potential to improve treatment outcomes and effectively address the complexities of modern oncology.

The basic premises of the N-of-1 tumor-tissue agnostic basket trials are: (i) initial dose reduction followed by intra-patients dose modification (i.e., individualized dosing) can be a safe way to administer previously unstudied combinations of drugs [8–10]; and (ii) one can evaluate the efficacy of the matching algorithm (e.g., the Matching Score) that reflects the degree of matching of drugs with the tumor, in determining efficacy, rather than the efficacy of individual therapeutic regimens in each tumor type [8–10].

We hope this paradigm will be understood and embraced more by all stakeholders including both industry and regulatory bodies. Indeed, N-of-1 basket trials can produce information on drug synergies and regimens active in individual patients efficiently. There may be similar patients that can be found and whose outcomes can be evaluated via large-scale interrogation of millions of computerized electronic medical records. Existing approved drugs used in combinations are the lowest hanging fruit for novel combination regimens as has been demonstrated by trials such as WINTHER [8], SPRING [21], and I-PREDICT [9, 10].

In 2024, the NCI has an innovative ComboMatch Trial that combines targeted agents with chemotherapy or other standard-of-care therapy. This is different from what WIN is pursuing as far as combinations of multiple agents that are individually tailored by the WIN MTB to the patients’ omics and AI analyses. The WIN Consortium views this N-of-1 approach as the future of oncology in general as has been emerging through studies in precision oncology published in the literature as cited above since 2010.

### Drug access for N-of-1 basket trials

Drug access is particularly helped by patient advocates and medication acquisition specialists. More challenging is drug availability for compounds that are investigational, mostly because of the numerous narrow eligibility criteria for most clinical trials. WIN Consortium leadership would argue it is very advantageous for industry to understand and support the N-of-1 precision oncology basket trials as a unique design with invaluable insight into benefit from active combinations with specific targeted agents or oncology treatment modalities, especially given the vast heterogeneity of cancer and emerging biomarkers [17, 18]. In the future, drug access, which can sometimes be a limitation, could be incorporated as an input into algorithms based on the patient’s geographical location to better address these challenges. The efforts of drug acquisition specialists and patient advocates can be incorporated into the management and conduct of precision oncology clinical trials.

### Next generation precision oncology trial design: World iterative next gen precision oncology study (WINGPO)

**Figure 5:**
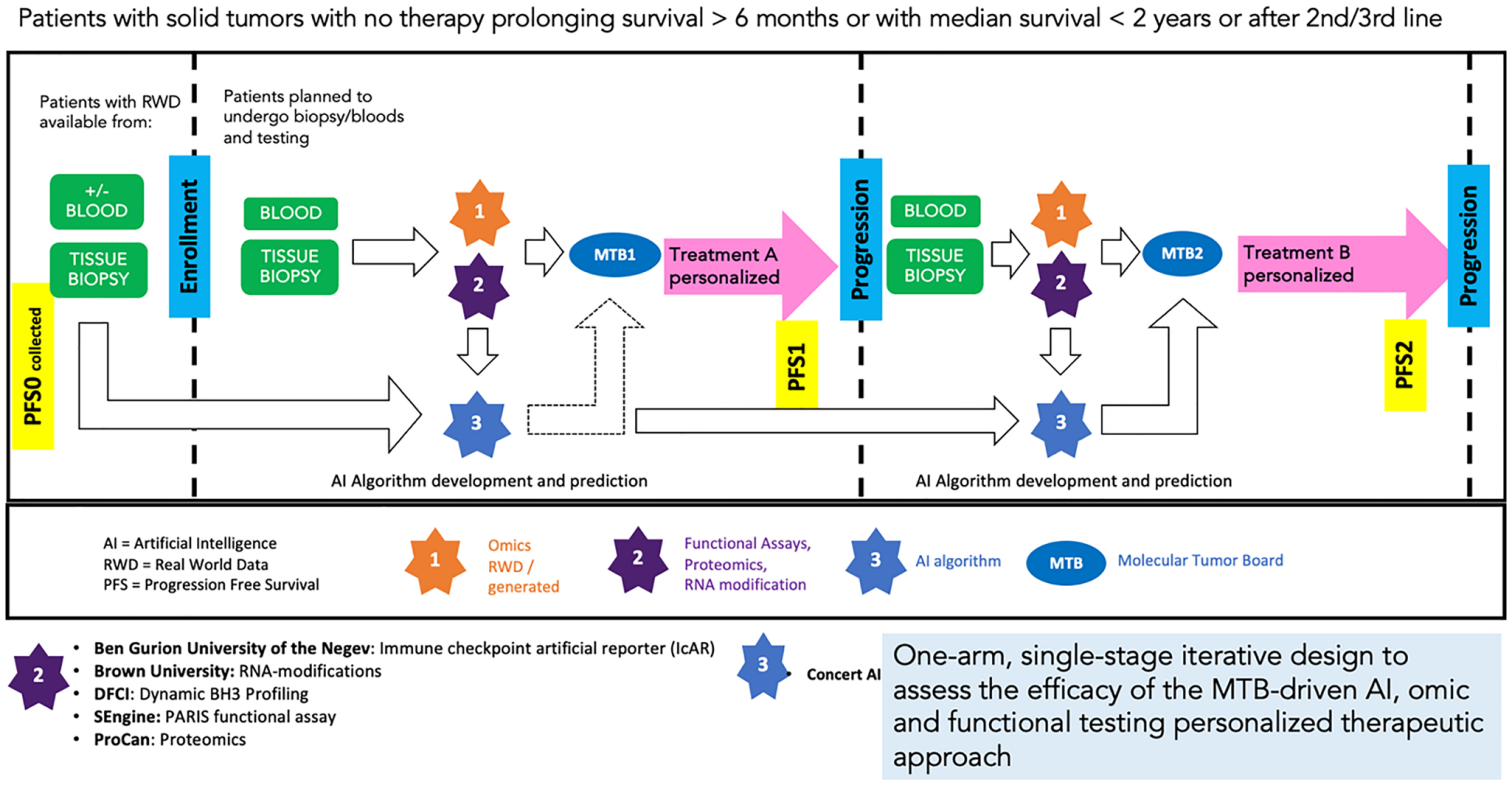
WINGPO N-of-1 precision oncology basket trial design in advanced cancer. WINGPO, guided by WIN expert stakeholders Molecular Tumor Board (MTB), enables optimal personalized treatment for patients with advanced cancer based on comprehensive range of investigations, development of AI algorithm(s) for treatment response prediction (agnostic of technology), understanding mechanisms and molecular basis for responders versus non-responders, understanding mechanisms of resistance to treatment, and analyses of outcomes and omics in different populations around the world.

WIN has conceptualized and evolved a next-generation clinical trial design in precision oncology known as World Iterative Next Gen Precision Oncology study (WINGPO) for patients with advanced cancer or with planned therapy that prolongs survival by less than 6 months or median survival of less than 2 years. WINGPO is an N-of-1 precision oncology navigational open basket study that provides cancer patients with optimal treatment selection (Figure 5). The drugs that might be considered will be FDA-approved drugs or combinations, drugs used off-label, or investigational agents subject to availability. Navigation to treatment clinical trials will also be considered. Combinations will be favored, if possible. The combinations that might be recommended are expected to be often suggested at a reduced dose to avoid toxicity as previously done in the I-PREDICT study [9, 10]. The best-matched therapy might not be available in the country or might not be accessible for a given patient. Drug availability will be taken into consideration by the study Molecular Tumor Board (MTB), which has an active role in the WINGPO design, and will be aided by drug-procurement specialists and advocacy groups. We believe that progress through communication with the FDA and industry will facilitate drug access and enable more N-of-1 basket trials in different patient settings in the future.

The design of the WINGPO study includes iterative (at trial baseline and at progressions) collection of agnostic real-world data (RWD) and generation of required genomic and transcriptomic profiling. There are also embedded in the study evolving novel omics, including functional precision medicine (FPM) assays that utilizes patient-derived organoids, proteomics, transcriptomics, RNA modification and immunomics.

WINGPO molecular profiling investigations rely on tumor and blood samples at different sequential time points allowing comprehensive assessment of the baseline tumor biology, and detection of changes in the tumor as it evolves under therapeutic pressure and relates to the patient survival outcomes.

Various machine learning and AI models are designed to be tested continuously as the data becomes available to reveal new treatment strategies and overcome resistance. Upon enrollment, the data collected will be reviewed by an MTB of experts that will provide several treatment options based on data available for personalized Treatment A, as illustrated in Figure 5. Upon progression under Treatment A, reiterative biopsy will be performed, and bloods will be collected for genomic and transcriptomic profiling and exploratory omics.

The MTB reconvenes to review updated data and recommend personalized Treatment B. AI algorithm(s) to predict and match therapeutic options are under consideration [30, 31]. Indeed, the iterative design permits biomarker validation in initial cohorts along with nomination to entry and therapy assignment in subsequent cohorts. The algorithm(s) predictions will be part of the data reviewed by the MTB to make their recommendations. The algorithm(s) will not be locked and will be constantly updated as new patients and new data are available. Similarly, investigations are not planned to be restricted and the trial plans to incorporate new tools and markers that may become available, given the quick evolution of the field.

The exploratory omics that are planned at this stage include:

1. Immune checkpoint artificial reporter (IcAR) technology (Ben-Gurion University of the Negev-BGU): IcAR technology will employ two methodologies based on fresh tumor samples to profile targeted therapy and one methodology, which could be employed to fixed/fresh samples, to evaluate immune checkpoint (IC) blockers ligands and specific treatments. Staining of tumor samples for the presence of ligands to IC receptors was not proven as a successful predictor for the PD1-PDL1 pathway. Moreover, TMB/MSI as a predictive biomarker for responses to IC therapy does not point to a specific type of IC blocker or differentiate the need for single agent versus combination therapy. IcAR technology [32] is a functional assay based on artificial reporters that can be applied to fixed/fresh tumor samples (primary/secondary growth) and to exosomes derived from blood of cancer-bearing patient and quantify the functional availability of IC ligands to a specific IC receptor (PD1, CTLA4, LAG3) and generate a score per each IC and the specific IC blocker drug to be applied (e.g. score per sample for functional ligands to PD1 IC + the effect of cemiplimab treatment). Therefore, IcAR technology can be used for prediction based on the original tumor sample. Yet, we could study the input from this technology during treatment by monitoring blood samples taken during the treatment for the functional availability of IC ligands on exosomes secreted into the bloodstream from the tumor microenvironment.
2. PARIS (Personalized, Aimed, Robotic, Informatics, Sequencing) Assay (SEngine): The initial success rate of the assay, defined as deriving organoid cultures and performing drug screens which passed QC steps was 72.5%. Most of the early screen failures (~20%) were due to inadequate quantity or viability of tumor cells at the time of receipt. These issues have been addressed through improved biopsy, shipping, and culture conditions and strict eligibility criteria (wash out of 3 weeks from chemotherapies). The current screen success rate is 92%. The PARIS assay identifies a drug with good to exceptional activity in 94.2% of patient derived tumor organoids (PDTOs) [33–39].
3. BH3 Profiling DBP (DFCI): The ability of DBP to predict response to therapy was demonstrated in several prospective murine studies, including GEMM and PDX; and several retrospective human studies [40–42].
4. Proteomics (ProCan): The proteomic technology allows the development of proteomic-based biomarkers (signatures) that are prognostic and predictive for response to treatment. This was shown in different types of solid tumors, including prostate cancer [43], pancreatic cancer [44], colorectal cancer [45], and melanoma [46], among others that are currently under development. This proteomic technology was also robust for developing diagnostic biomarkers [47, 48] to confirm tissue of origin and help diagnose cases of metastasis of unknown primary. ProCan’s technology also has the capability to mine the proteomic data to identify drug targets and assess response to treatment, integrating this with other multi-omic data [49]. This will allow the identification of the pathways associated with response and resistance to specific drugs as well as the identification of novel drug targets that can be used for patients who run out of treatment options.
5. RNA-modifications (Brown University): RNA-modifications remain largely unexplored [50, 51]. Global analysis of modified nucleosides will be performed using an Exploris 240 Orbitrap Liquid Chromatography Mass Spectrometer (LC-MS) recently purchased by the Brown RNA Center. The analysis does not require intact RNA. Global nucleoside analysis will provide patterns of modified nucleosides to serve as signatures for diagnosis and also for comparing responses to treatment, where nucleoside patterns from normal tissue may serve as the baseline. Currently 100,000 to 1 million cells can be used but protocols are under development to analyze 1000 cells for RNA modifications from tRNA and ribosomes.

The one-arm, single-stage design will be used to assess the preliminary efficacy of the study MTB-driven personalized therapeutic approach. The primary efficacy analyses will be based on the ratio between the progression time on personalized therapy to the progression time on prior therapy. The design of the study as in other N-of-1 trials such as WINTHER, poses the question of the use of the PFS comparison to the PFS of the prior therapy when the latter is prior to inclusion in the study. In this case, the so-called PFS0 remains uncontrolled and may be overstated as radiologic assessments done as part of standard-of-care are usually done at longer intervals than in a clinical trial (3 months vs. 2 months). To limit this substantial hurdle, either the prior treatment (PFS0) should be included in the study (which would be by far the best solution) or alternatively the study treatment assessments (PFS1 and subsequent) should be done at similar intervals than the PFS0, according to institutional calendar. But more generally, the issue of timing of first treatment assessment and regular follow up to monitor disease and identify early progression should be reconsidered as new surveillance tools such as circulating tumor DNA (ctDNA) based on non-invasive blood investigations become available [52]. Predicting responses to targeted therapies with ctDNA is already feasible as early as the first week, using urine or blood samples. Immunotherapies, however, may require a longer timeframe to gauge response via blood-based ctDNA [53, 54]. Waiting 2 or 3 months to assess whether a treatment is effective seems a long time that can potentially harm the patient. Depending upon therapy selected, leaving a patient under a treatment for several weeks where there is the possibility of assessing early response and more importantly early non-response can lead to the patient losing a window of opportunity for efficacy of another treatment. The WINGPO trial will aim to include regular monitoring of the disease with first assessment by ctDNA after the first week of treatment.

### Precision oncology in the neoadjuvant and adjuvant cancer settings

Earlier-stage therapy of malignant tumors has provided a path towards cure and improved patient survival. A major goal in the practice of oncology is early detection and accurate diagnosis of cancer that is potentially curable. With the advent of targeted therapies and progress in precision oncology, it has become very important to determine the impact of these therapies as it relates to cure in earlier cancer stages. Recent landmark studies have begun to demonstrate the value of neoadjuvant immunotherapy in melanoma [55, 56] and colorectal cancer [57].

The FDA’s Project Frontrunner [58] seeks to “encourage drug sponsors to consider when it may be appropriate to develop and seek approval of cancer drugs for advanced or metastatic disease, in an earlier clinical setting…”. A number of advantages have been outlined by the FDA including earlier access to therapies, improved assessment of drug effects, comparisons of novel therapies with standard of care, and potential to improve treatments in the frontline setting.

**Figure 6:**
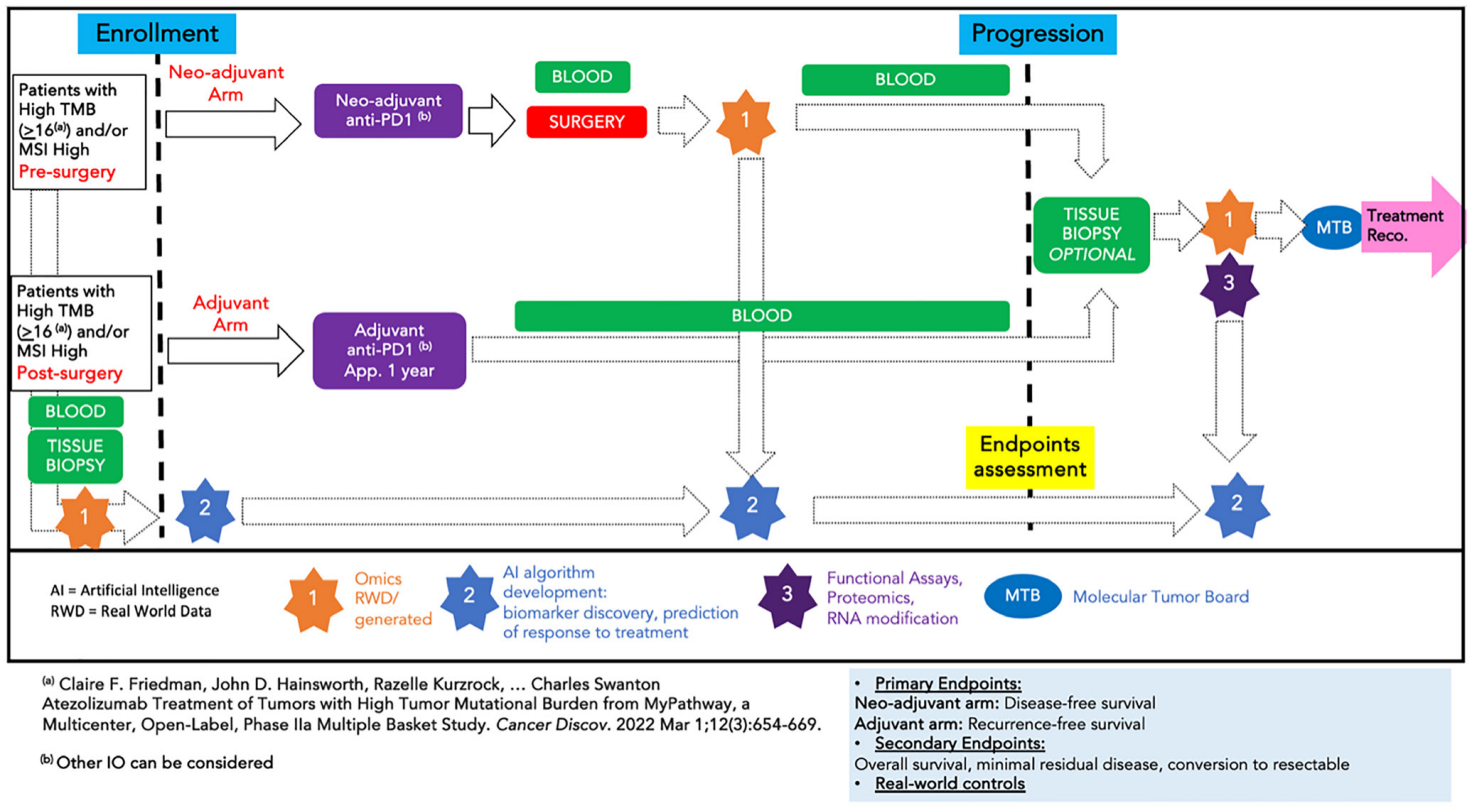
Randomization to Neo-adjuvant or Adjuvant Immunotherapy for Patients with solid tumors with TMB high (>16(^a^)) or MSI high (NEOVADJ-TMB-IO). N-of-1 clinical trial schema for neoadjuvant versus adjuvant immunotherapy in the treatment of early-stage cancer. Symbols are as described in Figure 5.

The WIN Consortium developed in 2024 a trial schema to compare neoadjuvant versus adjuvant immunotherapy at earlier cancer stages as depicted in Figure 6. The proposed design, which integrates AI tools alongside the expertise of MTB specialists which have the final authority, was developed to gather important data about the utility of this approach and its potential to enhance outcomes compared to real-world data controls. This does not necessarily suggest that standard-of-care should be changed in adjuvant or neoadjuvant settings but rather to compare them and try to improve the available therapies based on MTB input. This type of data with similar cohorts of patients will be critical evidence for any superiority of personalized treatment as compared to contemporary real-world data or matched control patients within actual trials. AI is not selecting therapies but rather assisting with analysis for the MTB that recommends optimal therapy. The MTB could for example provide options for stage 3 colorectal cancer or oligometastatic disease such as FOLFOX or immunotherapy based on MSI status -/+ targeted therapy such as a PI3K inhibitor or an NTRK or Her2 blocker or a KRAS drug -/+ anti-EGFR. The statistical design and power of the precision oncology trials will be critical for demonstrating superiority and not just non-inferiority, which can be done convincingly with contemporary real-world or even trial controls.

Personalized combination treatment based on a cancers’ drivers along with personalized vaccine approaches in the adjuvant setting should be investigated more in a tissue agnostic manner. The data should drive the practice, but we won’t know if we don’t try.

### Mutations across borders

The WIN consortium (WIN) endeavours to systematically map driver mutation variations across diverse cancer types and countries. Capitalizing on WIN’s global network, the study proposes to establish a database of driver mutation frequencies. By gathering de-identified data from WIN’s sites around the world, we plan to compile a robust dataset detailing the frequency of specific mutations across various cancer types, stages, genders, ethnicities, from different diagnostic technologies, and tissue sources from which the diagnosis was derived. This data will be utilized to generate similarity trees, illustrating the genetic similarities and differences between countries. This will aid clinicians to interpret cancer research, allowing them to apply findings from genetically similar populations to their local patients more effectively. Additionally, artificial intelligence (AI) algorithms will be employed to identify enriched features, to investigate the association between feature measurements collected and literature, and to assess the predictive potential of these features. It will guide us towards the identification of novel risk factors, mutation patterns, and potential therapeutic targets and their prevalence in specific geographic areas. Based upon the initial data collected, questionnaires will be developed to the attention of WIN sites to query on genetic and environmental factors, treatment responses, and patient outcomes, if possible. This advanced database will enable answering an array of scientific questions and therefore facilitate research and opportunities for clinical application. This unique resource of genetic and environmental cancer data will eventually be made available to the vast community. In summary, this international collaboration seeks to create through systematic mapping of driver mutations and AI, a globally integrated framework for cancer mutation analysis. It is expected to help clinicians across the world make sense of international research, guide them in selecting treatments tailored to their individual patients and improve outcomes.

### Iterative innovation

A cornerstone the WIN Consortium upholds involves innovation in the field of precision oncology. This has motivated the like-minded member organizations since its founding and continues to the present day. There are numerous technological advances in candidate biomarkers, omics, and AI tools. The WIN clinical trial designs allow for real-world testing of novel measurements and tools that can then be advanced into consideration by the MTB in later cohorts. The iterative nature of implementing innovations through continuous learning will always keep WIN at the forefront of advances that can serve patients. Thus, ‘iterative innovation’ represents a key aspect of the way the WIN Consortium remains focused on scientific discoveries and their translation.

### Challenges with FDA cancer drug approvals

In addition to the well-known issue of drug access in general in oncology, and more specifically in the setting of innovative clinical trials, there are additional interrelated challenges in precision oncology. The FDA historically has not approved cancer therapies on the basis of N-of-1 combination therapy clinical trials. While tissue agnostic approvals have occurred since 2017 with the use of immunotherapy to treat mismatch repair-deficient cancer, N-of-1 combination therapies based on N-of-1 trials have not yet led to specific approvals. As data accumulates, the FDA and other regulatory oversight bodies will need to be prepared to evaluate aggregated data involving similar cohorts of patients who are shown to benefit in N-of-1 basket trials. An openness to look at such data to bring effective combinations to patients at the earliest time is advantageous for patients, caregivers, payors, governments and drug manufacturers.

An openness by the FDA to consider for drug approval N-of-1 combination therapy clinical trial evidence as has been accumulating for 15 years in the field, for new indications, will encourage more effort to gain additional post-approval results. Importantly, the way FDA and other regulatory bodies view N-of-1 basket trials as far as potential drug approval in new indications should stimulate drug manufacturers to more enthusiastically support and participate in N-of-1 trials. There would certainly be ample opportunity in this realm for real-world control arms. The potential for drug combinations in N-of-1 basket trials includes (or should include) all possible modalities in oncology, small molecules, biologics, immunotherapy, on-label use, off-label use, drug repurposing, and investigational use.

As there is already a science to safely dose novel drug combinations, the implementation of a large number of N-of-1 trials is not only feasible but somewhat overdue. The efficiency of N-of-1 trials in finding efficacy signals of novel cancer therapy combinations dictates that the field of oncology get beyond a traditional reluctance to go beyond conventional clinical trial methodologies. One of the major regulatory challenges involves the approval of a companion diagnostic that can guide selection of treatment in the n=1 setting so biomarker development will need to occur alongside the machine learning algorithm and the drug/drug combination in parallel. The FDA can play an important role in catalyzing what some would consider a revolutionary change to accelerate progress in oncology.

### Challenges with industry

Despite clear pathways established by regulatory agencies [59], industry [51] and other stakeholders remain reluctant to consider N-of-1 trials. In part, this is because they are a new paradigm driven by what powerful new technology has revealed, e.g., that each patient’s tumor is complex and unique. Beyond simply meeting the needs and expressed wishes of the broader patient community, N-of-1 trials have an opportunity to provide early proof-of-principle including on-target efficacy, broader efficacy and safety data in underrepresented populations, and establish robust translational science platforms. Most importantly, these are not “single patient” trials. Although each patient may be treated in a unique manner because their tumor biology is unique, specific matching algorithms can be developed and the robustness of those algorithms assessed [8, 10] making them applicable across large numbers if not most cancer patients.

**Figure 7:**
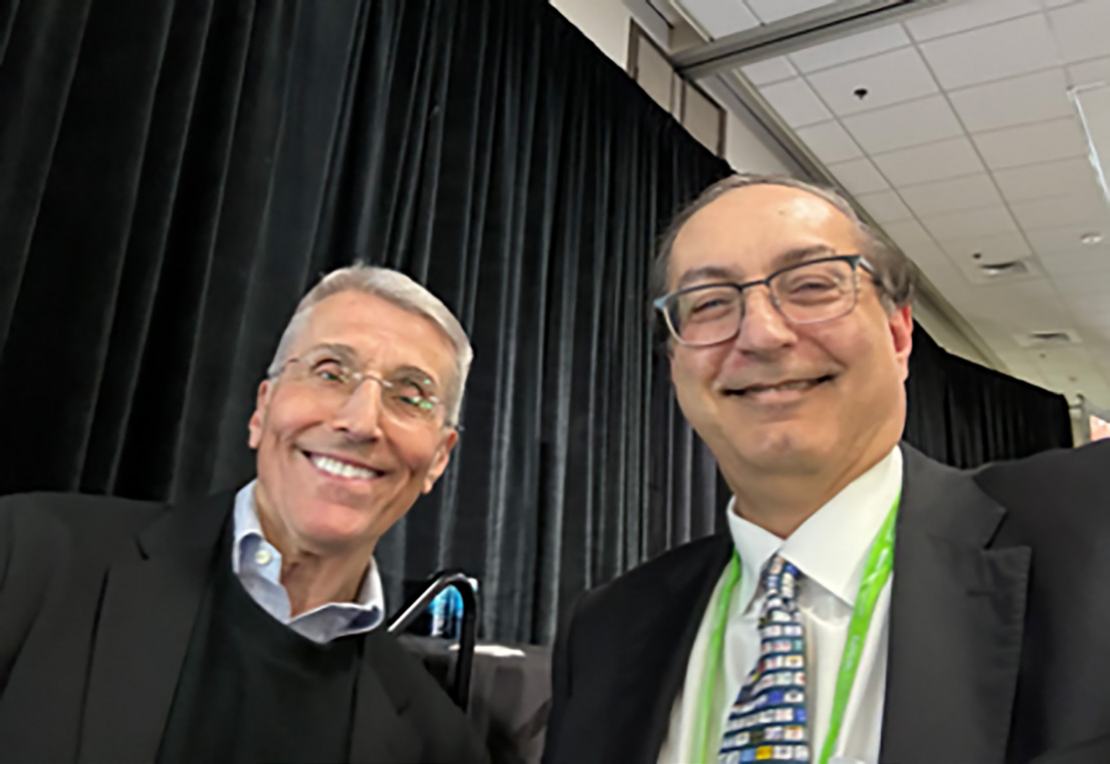
On April 5, 2024, WIN Consortium Chair El-Deiry meets Dr. Richard Pazdur, Director of FDA’s Oncology Center of Excellence.

W.S.E-D. had the privilege of meeting FDA’s Dr. Richard Pazdur (Figure 7) after Dr. Pazdur gave a fireside chat with numerous pearls. Dr. Pazdur communicated the importance of getting drug approvals right as far as safety and efficacy, the importance of survival outcomes, confirmatory trials, the importance of drug dosing, conflict of interest, level playing field, and the resource intensiveness of drug withdrawals after initial approval. Dr. Pazdur communicated that it is important to simplify clinical trial designs in oncology as trials have become very complex trying to ask and answer too many things at once. When they briefly met, Dr. El-Deiry mentioned the WIN Consortium and efforts to advance the field of Oncology through N-of-1 clinical trials.

The history of oncology for the last 80+ years has been combination therapy with chemotherapy using drugs with non-overlapping toxicities. With so many advances in recent decades and knowledge about toxicities, the founding principles of oncology come back into play in the third decade of the 21st century. It can similarly be argued that post-approval, N-of-1 trials have an important role to play in finding additional indications based on favorable results from combination therapies in similar cohorts of patients.

Clinical trialists in 2024 are very familiar with the exceedingly complex early-phase trial designs with numerous combination arms that have monopolized patient populations without optimizing potential benefits. It could be argued that the availability of N-of-1 clinical trials offer an important aspect to the risk-benefit calculation in clinical trials in oncology. As to many oncologists, N-of-1 therapies based on omics, AI algorithms, and MTBs is the future of oncology. The real question is when does this happen in the mainstream? It is worth considering that buy in and participation by industry can play an important role in the evolution of improved patient care in oncology by providing individualized therapies in a personalized way for patients.

Dr. Kurzrock has pointed out that the FDA has found ways to fast track/accelerate approvals, for biomarker-driven therapies, requiring just a small number of patients (sometimes ~30 to 100 patients) for approval, providing that response rates are high and durable [60–62]. This is an important departure from traditional randomized trials of unselected patients that look for small improvements and require very large numbers of participants as well as years to perform. Finally, as shown by tumor-agnostic approvals [63–66], rare biomarkers in a single disease can become quite a large market once the targeted drug is applied to all cancers bearing the biomarker. Most importantly, giving the right drug(s) to patients, rather than combinations that include the right drug but also additional drugs that might not be of use to that patient (but add toxicity), is simply the right thing to do for patients with cancer.

## CONCLUSIONS

Our hypothesis is that comprehensive omics in tissues and blood as well as functional precision medicine, AI algorithms and MTB-guided N-of-1 drug selection will bring best-in-the-world iterative treatment options for patients with malignancies to prolong survival and ultimately save money and lives by treating the right patients with the right drugs/combinations. The WIN Consortium approach enables understanding of the molecular basis for responders versus non-responders, elucidate mechanisms of resistance to treatment, and analyze outcomes *vis a vis* omics in different populations around the world.

To bring precision oncology studies to fruition, whilst grant application opportunities are being pursued, interaction and collaboration with pharmaceutical companies, large and smaller biotechnology, and AI and genomics/omics companies are sought and will allow the WIN consortium to maximize impact through innovation and collaboration. Of particular importance is the patient voice and inclusion of patient advocacy groups in WIN as they push to accelerate the future of precision oncology and work with other stakeholders to address the challenges that are faced. The MTB is providing a forum for discussion of complex patient cases with omics, and these are in process of publication for educational purposes.

The WIN Consortium is in the process of establishing a one-year self-funded WIN International Fellowship in Precision Medicine under the leadership of Drs. Kurzrock and El-Deiry. Candidates can be of any stage of training, faculty or industry, can have MD or PhD degree or be in training for MD or PhD degree. Opportunities include visit WIN member organizations for 3 months to one year if desired, self-funded, completion of at least one manuscript and/or at least one clinical trial written, submission and presentation of a WIN abstract at >1 WIN meeting and/or a national/international meeting, involvement and assistance with the WIN MTB as “mentern” —including write ups for publication, opportunity to serve on at least one WIN committee as trainee member. Since WIN includes a broad away of organizations, such as academic, industry, patient advocacy, etc., WIN consortium fellows have opportunity to work with one or more of these stakeholders in the precision oncology space. Fellows can assist with WIN consortium social media interviews and news. Successful fellows will receive a Certificate of Completion from the training at the end of the experience.

With our ground-breaking spirit and expertise in complex investigator-led studies, our global footprint and nimble organization, we can foster collaborations and alliances, leverage and maximize expertise, ensure equitable distribution of resources, and promote data sharing whilst always keeping the patients at the center of everything we do.
